# Normal umbilical artery doppler values in 18–22 week old fetuses with single umbilical artery

**DOI:** 10.1038/s41598-023-37691-z

**Published:** 2023-06-28

**Authors:** Omer Kazci, Sonay Aydin, Erdem Fatihoglu, Oğuzhan Tokur, Suzan Bahadir, Erdal Karavas, Mecit Kantarci

**Affiliations:** 1 Department of Radiology, University of Health Sciences Ankara Training and Research Hospital, Hacettepe Mh. Ulucanlar Cd. No: 89, 06230 Altındağ/Ankara, Turkey; 2 Department of Radiology, Erzincan University, Erzincan, Turkey; 3 Baskent University, Antalya, Turkey; 4 Department of Radiology, Bandirma Onyedi Eylul University, Balikesir, Turkey; 5 Department of Radiology, Ankara Training and Research Hospital, Ankara, Turkey

**Keywords:** Medical research, Ultrasonography, Intrauterine growth

## Abstract

Umbilical cord with a single umbilical artery (SUA) can carry twice the blood volume of a three-vessel cord (TVC). So, the normal hemodynamics of the fetuses with SUA was different from those with TVC. Furthermore, structural abnormalities, fetal aneuploidy, and intrinsic growth retardation may be associated with the presence of a SUA. In order to evaluate these patients, intermittent doppler measurements have been suggested. From this point, we aimed to determine the CDUS flow parameters in SUA cases and to demonstrate that these flow parameters are different from the TVC parameters. Ultrasound (US) examinations were performed in the 18–22 weeks of gestation during routine fetal anatomy screening. Resistance index (RI), Pulsatility index (PI), and S/D: systole to diastole ratio values were measured. The samples were taken from the proximal, mid-portion, and distal of the umbilical cord. In addition to Doppler Ultrasound values, AC and estimated fetal weight (EFW) values were also recorded. The study included 167 pregnant women, 86 of whom were study group with SUA and 81 were control group with TVC. The measurements of RI, PI, and S/D at all three levels were significantly lower in the SUA group compared to the TVC group. The resistance in the UA of fetuses with SUA is lower than in fetuses with TVC. The resistance in the UA of fetuses with SUA decreases from the fetal end to the placental end. Knowing the normal values for fetuses with SUA might provide a better and more reliable Doppler Ultrasoundassessment.

## Introduction

Two arteries and one vein compose the umbilical cord and it is surrounded by Wharton’s jelly. Single umbilical artery (SUA), the most common anomaly of the umbilical cord, and is observed 0.5–5% of all pregnancies^1^. SUA frequently has been associated with intrauterine growth retardation (IUGR), congenital/chromosomal abnormalities, prematurity, and increased perinatal mortality^2,3^. Almost all placentas have Hyrtl anastomosis close to umbilical cord insertion site. This anastomosis contributes to avoid umbilical cord blood flow deficiency between two umbilical cord arteries during pregnancy. Even if somewhat accidental external force to the umbilical cord affects to these flows, this anastomosis and umbilical arterial resistance can adjust these flows. So, even in case SUA, one dilatated artery and anastomosis can sufficiently send blood flow into the placenta^4^.

Fetuses with SUA are commonly considered at increased risk for perinatal complications. Umbilical arterial (UA) Doppler assessment is frequently used to assess fetal well-being, especially in high-risk pregnancies. It has been shown to reduce perinatal mortality and morbidity especially in high-risk patients^5^.

The prevalence of a single umbilical artery in all fetuses is 1%^6^. Some recent studies have shown that the umbilical cord with SUA is able to carry twice the blood volume of a three-vessel cord with two umbilical arteries and meet the required flow^7^. As a result, the normal hemodynamics of the fetuses with SUA was different from those with normal UA, and previously defined normal parameters might not be suitable for fetuses with SUA. There have been a very limited number of studies in the literature^7,8^ to examine normal color Doppler ultrasound (CDUS) values of the SUA cases due to low prevalence of SUA. There are some studies suggest that the presence of a single umbilical artery might be associated with structural abnormalities, fetal aneuploidy, and intrinsic growth retardation and also intermittent biometric and doppler measures of these patients have been suggested to evaluate^8–11^. From this point, we aimed to determine Doppler ultrasound flow parameters in SUA cases and to demonstrate that these flow parameters are different from the three-vesse cord parameters.

## Materials and methods

**Figure 1:**
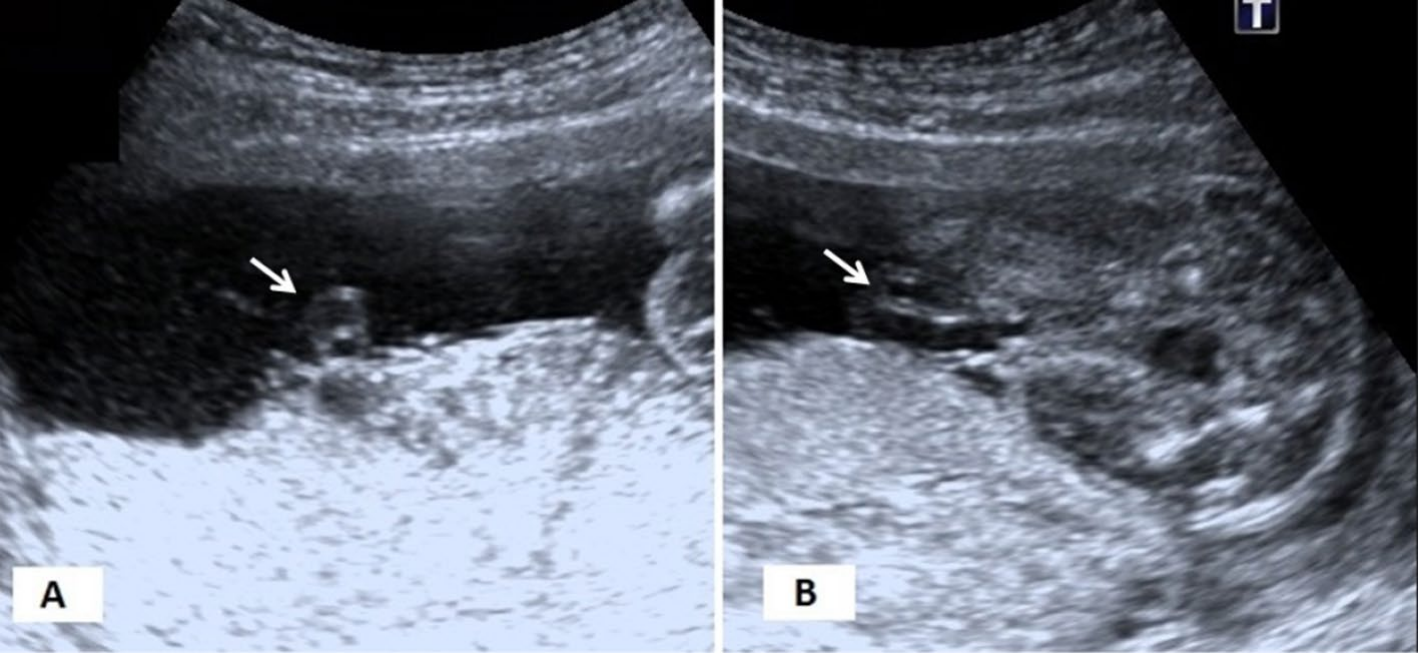
Ultrasound diagnosis of the single umbilical artery is usually performed by evaluation of vessels around the fetal bladder rather than by evaluation of umbilical vessels on (**A**) axial and (**B**) longitudinal plane.

The study was conducted between January 2018 and May 2020. Ultrasound (US) examinations were performed in the 18–22 weeks of gestation during routine fetal anatomy screening. All the pregnant who were referred to the radiology department for detailed morphological abnormality scan were examined during the study period. Then, these pregnant were followed‑up until the labor to get information about pregnancy outcome. If any of the following conditions were diagnosed during the follow-up period, patients were excluded from the study; maternal diseases or diabetes, hypertension or preeclampsia, drug abuse, fetal anomaly, IUGR, multiple fetuses, perinatal death, placental abnormalities. Fetuses with SUA have constituted the study group (Fig. 1). Pregnant with a 3-vessel cord (TVC) who were accepted to participate in the study constituted the control group.

**Figure 2:**
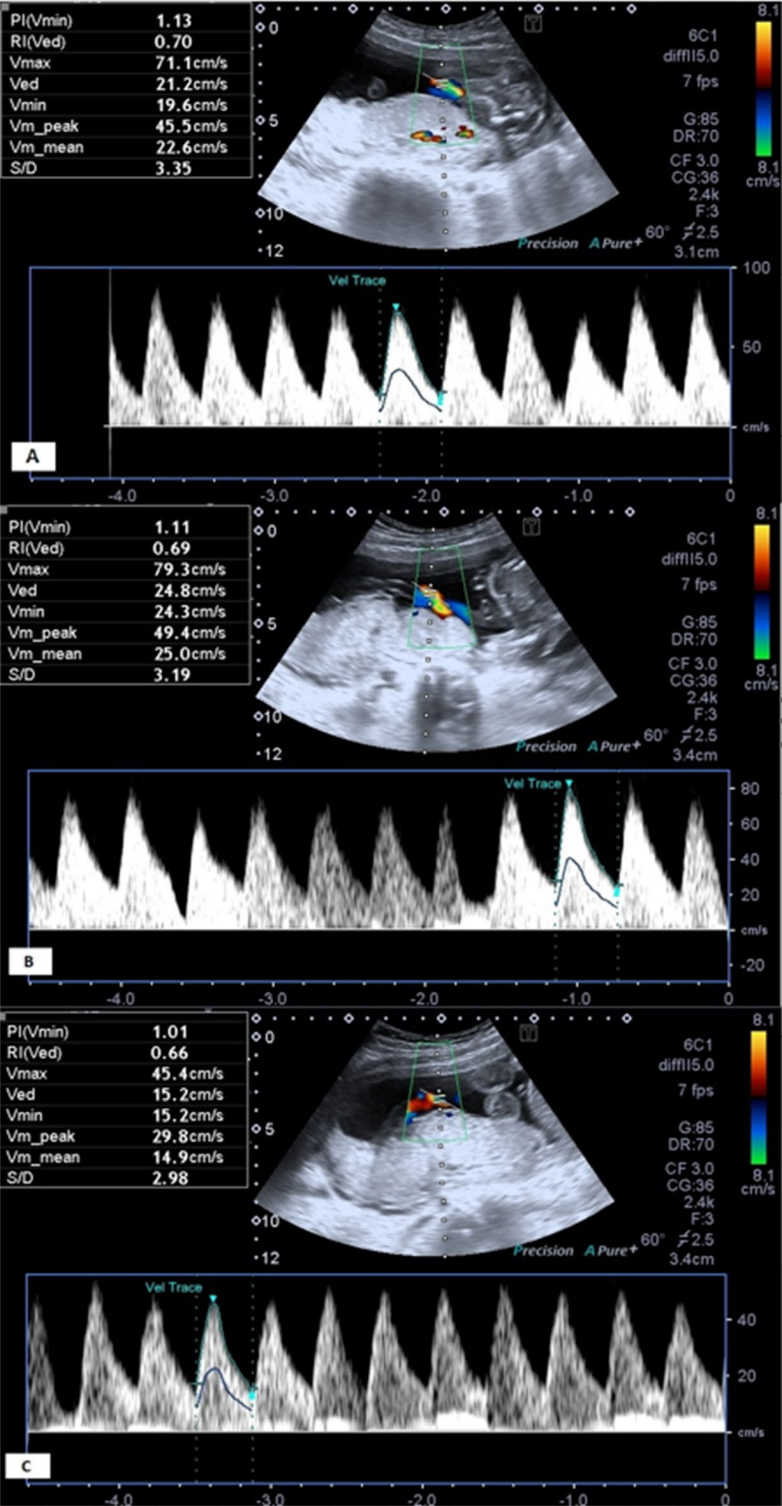
Flow patterns and Doppler ultrasound indexes of the umbilical artery from the fetal end (**A**), mid-portion/free loop (**B**), and the placental end (**C**).

**Figure 3:**
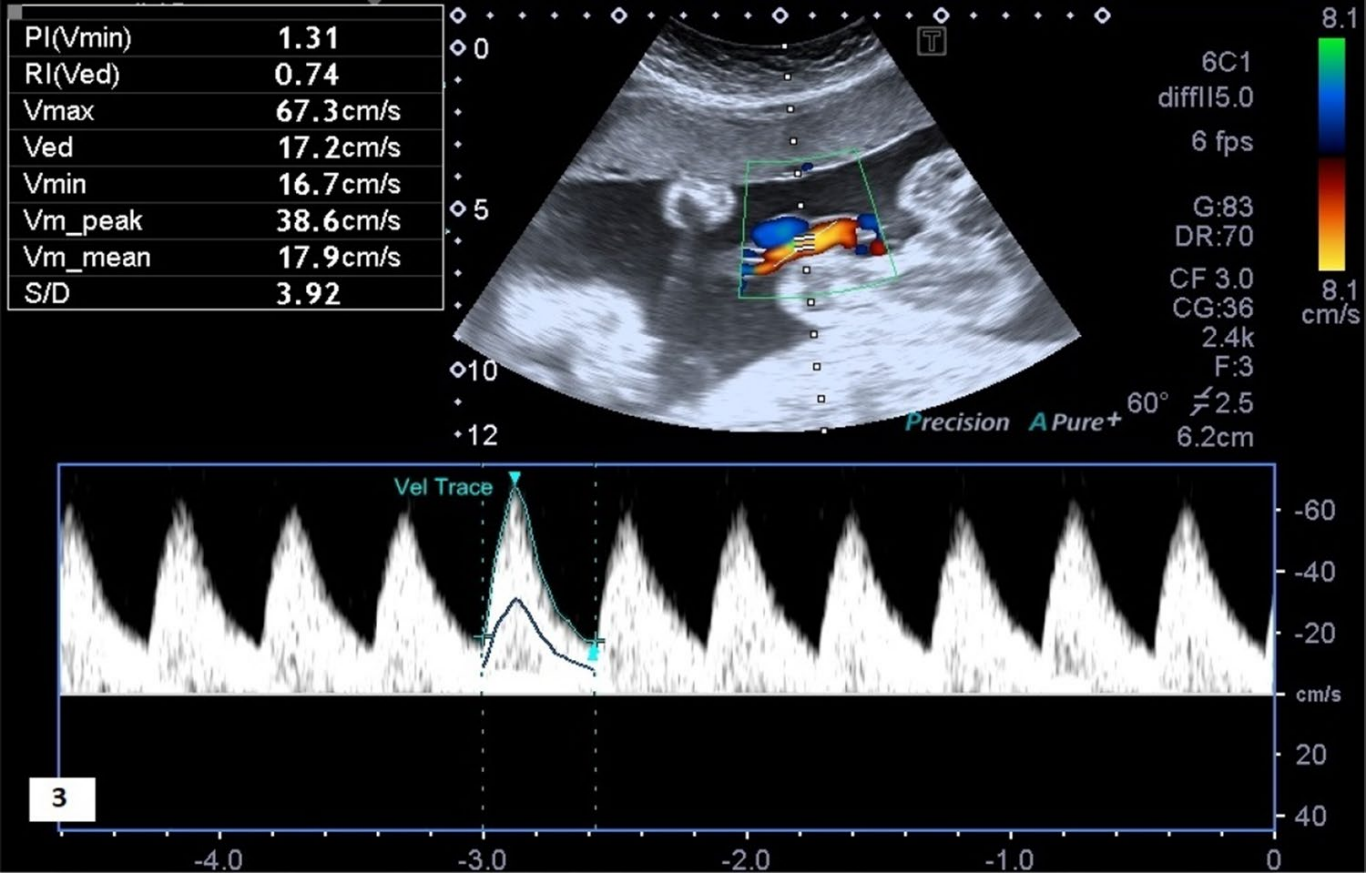
Flow pattern and Doppler ultrasound indexes of the umbilical artery in a three-vessel cord fetus.

All patients were examined with a gray-scale, color, and pulsed Doppler ultrasound machine (Toshiba, Xario) with a 3.5 MHz convex transducer. UA Doppler ultrasound parameters were acquired by using the standardized image which was previously defined^12^ for abdominal circumference measurements. Doppler indices is with the direction of the ultrasound beam being parallel to the longitudinal axis of the UA or forming an angle of less than 30. The Doppler US was performed at standard angles, between + 45° and − 15° and all measurements were made During fetal apnea and absence of fetal movements and in the supine position of pregnant, by two experienced (8 and 15 years of experience) radiologists. Resistance index (RI), Pulsatility index (PI), and S/D: systole to diastole ratio values were measured. The samples were taken from the proximal (1/3 fetal part), mid-portion, and distal (1/3 placental part) of the umbilical cord (Figs. 2 and 3). In addition to CDUS values, AC and estimated fetal weight (EFW) values were also recorded. Doppler measurements were taken in a free loop of the umbilical cord, when at least a minimum of three repetitive waveforms were documented Measurement averages of the two radiologists were accepted as final data.

### Statistical analyses

Data were analyzed using the IBM Statistical Package for Social Sciences v25 for Windows (IBM SPSS Inc., Chicago, IL). The normal distribution of the data was evaluated with the Kolmogorov–Smirnov test with Lillefors significance correction. Levene’s tets was used to evaluate the homogeneity of the variances. Nomality of the data was also checked with skewness and kurtosis. Numerical variables with normal distribution were shown as mean ± standard deviation [SD]. Categorical variables were shown as numbers and percentages. For the comparison of numerical variables between two groups, student t-test was used. We compared categorical variables between two groups by using the Pearson Chi-Square test. For repeated measures ANOVA test was used to analyze the difference of the parameters between three different locations of the umbilical artery, Tukey test was applied for post-hoc analyzes. Categorical correlation analysis (Cohen’s kappa analysis) was performed to test interobserver variability.

A two-tailed value of p < 0.05 was considered statistically significant.

### Ethical approval

All procedures performed in studies involving human participants were in accordance with the ethical standards of the institutional and/or national research committee and with the 1964 Helsinki declaration and its later amendments or comparable ethical standards. This cross-sectional study was conducted after obtaining approval from the Ethics Committee of the Erzican Binali Yildirim University hospital (EBYU-KAEK_ 2018-01-04). All included patients provided their written informed consent for participating in the study.

## Results

The study included 167 pregnant women, 86 of whom were study group with SUA and 81 were control group with TVC. The mean age of the pregnant women was 28.20 ± 5.49 (min–max;18–40).

The mean age was 29.77 ± 5.05. in the SUA group and 28.54 ± 5.48 in the control group. There was no statistically significant difference between the mean ages (p = 0.07).

The mean gestational week of the all study population was 20.1 ± 1.4 weeks (min–max; 18–23 weeks). The mean gestational week was 20.1 ± 1.4 in the SUA group and 20 ± 1.4 in the control group.

Study and control group results were similar in terms of the mean gestational week (p = 0.61).

77 (46.1%) of the fetuses were male and 90 (53.9%) were female, and the distribution of the fetus genders by groups is presented in Table 1. There was no statistically significant difference between the two groups regarding the distribution of fetal gender (p = 0.91).

**Table 1:** The distribution of the fetal gender by study and control groups.

Fetal gender	Single UA	Control
Male	40	37
Female	46	44
Total	86	81
Mean AC value	152.95 ± 16.39 mm	155.16 ± 15.87 mm
Mean EFW value	367.01 ± 99.19 mm	374.30 ± 75.81 mm
Mean neonatal weight at birth	3010.43 ± 85.11 gr	3087.03 ± 48.91 gr

*AC* abdominal circumference, *EFW* estimated fetal weight.

The mean AC value of the population is 152.22 ± 16.14 mm. There was no significant difference between the mean AC values in both groups (p = 0.55). The mean estimated fetal weight (EFW) value of the population is 370.54 ± 88.42 mm. There was no significant difference between the average EFW values in both groups (p = 0.34). The mean neonatal weight at birth was 3047.56 ± 68.14 gr., neonatal weight was similar between SUA and control groups (p = 0.56). The AC, EFW and neonatal weight at birth values of the subgroups can be found in detain in Table 1.

Mean and 3–97 percentile values of RI, PI, and S/D are shown in Table 2 for both groups. The measurements of all parameters at all three levels were significantly lower in the SUA group compared to the control group.

**Table 2:** Mean ± standart deviation and 3–97 percentile values of RI, PI and SD by study and control groups.

Parameters	SUA	Control	P value
RI			
Fetal end	0.63 ± 0.08 (0.50–0.80)	0.74 ± 0.07 (0.60–0.90)	p < 0.001
Mid-portion	0.62 ± 0.06 (0.47–0.71)	0.70 ± 0.06 (0.58–0.87)	p < 0.001
Placental end	0.60 ± 0.09 (0.43–0.68)	0.68 ± 0.06 (0.55–0.83)	p < 0.001
PI			
Fetal end	0.94 ± 0.12 (0.80–1.20)	1.15 ± 0.13 (1–1.5)	p < 0.001
Mid-portion	0.93 ± 0.13 (0.76–1.17)	1.10 ± 0.11 (0.9–1.3)	p < 0.001
Placental end	0.92 ± 0.13 (0.74–1.13)	1.07 ± 0.1 (0.82–1.1)	p < 0.001
S/D			
Fetal end	3.06 ± 0.47 (2.36–4)	3.98 ± 0.99 (2.84–6.16)	p < 0.001
Mid-portion	2.91 ± 0.57 (2.20–3.87)	3.79 ± 0.88 (2.71–6)	p < 0.001
Placental end	2.79 ± 0.62 (2.06–3.64)	3.70 ± 0.87 (2.63–5.85)	p < 0.001

*RI* resistive index, *PI* pulsatility index, *S/D* systole to diastole ratio, *SUA* single umbilical artery.

Mean RI, PI and S/D values decreased significantly from fetus to placenta (proximal to distal) in both SUA and control groups (p < 0.001).

Interobserver variability was good for all three measures (ĸ values between 0.77–0.80, good).

## Discussion

In this study, we found that fetuses with SUA have different normal Doppler ultrasound index values in comparison with fetuses with TVC. RI, PI, and S/D values were significantly lower in fetuses with SUA. These values also significantly decrease as the location of the measurement nears the placenta.

UA Doppler ultrasounds are commonly associated with fetal well-being assessment, especially for the evaluation of small for gestational age (SGA) fetuses. Despite their wide use, normal ranges for UA Doppler ultrasound values were generally defined for TVCs. Normative values for SUA were only defined in a very limited number of studies with small population numbers^6,7^.

We studied two groups (SUA and control), which were very similar other than UA characteristics. The AC, EFW values, and distribution of the fetal gender were similar between the groups. As previously stated, AC and EFW values might affect the UA Doppler ultrasound values^14^. We have managed to neutralize such an effect, and we believe that this can increase the reliability of the presented data. In line with the current study, previous studies^6,7^ also utilized SUA and control groups with similar EFWs. Different from the previous studies, we also offered information about fetal gender; SUA and control groups had similar rates of fetal gender. It can be inferred that the difference in UA Doppler ultrasound values was not related to or depended on the fetal gender.

It was stated previously that the highest impedance was found at the fetal end of the UA, and the changes in the CDUS indexes are likely to be seen at the fetal end first. Due to the difficulty of performing measurements at the fetal end, especially in growth-restricted fetuses, measurement in the mid-portion or free loop was stated to be acceptable^15^. In the previous studies, the free loop^6,7^ and fetal end^8^ were preferred for the Doppler ultrasound measurements; as far as we know, there are no previous studies to examine all three parts of the UA in SUA cases. According to our results, the trend of the impedance within the UA was the same for both SUA cases and the fetuses with TVC.

The mean RI, PI, and S/D values of the control group (normal fetuses with TVC) were within the previously defined normal values^16,17^, which confirms the value and strength of the control group and the defined differences.

Previous studies concluded that the RI, PI, and S/D values of the SUA cases were lower than those of the fetuses with TVC^7,8^. Our results confirmed the previously presented data; we have shown that RI, PI, and S/D values significantly decreased in all three parts of the UA in the fetuses with SUA. Some strengths of this study are worth emphasizing. According to our knowledge, this is the first study to provide second-trimester CDUS norms for SUA cases. The current study also confirms that the previously defined decrease in RI, PI, and S/D values was valid not only for third-trimester examinations but also for the second trimester. The interobserver variability of the presented data was good for all of the measurements, which can increase the reliability of the normal values. Also, the previously presented data belonged to third-trimester pregnancies^8,9^. In the literature, defined normative values include both the second and the third trimesters^16,17^. As far as we know, this is the first study to offer normative second-trimester Doppler ultrasound values for SUA cases. The current study also confirms that the previously defined decrease in RI, PI, and S/D values was valid not only for third-trimester examinations but also for the second trimester. The interobserver variability of the presented data was good for all of the measurements, which can increase the reliability of the normal values.

This study has some limitations worth mentioning. First of all, even with the largest population number, the study might offer more reliable results with more participants. We have performed the study with an experienced radiologist; we do not know if the values change with the level of experience. We have studied with a pregnant woman who was healthy apart from having an SUA. Studying the fetuses with SUA and other pathologies, such as SGA, might increase the reliability of the data. We have studied pregnant women in the 18–23 gestational weeks, and we have presented normal values for the second trimester. Examining each week separately might increase the reliability and practicality of the results.

To conclude, the resistance in the UA of fetuses with SUA is lower than in fetuses with TVC. The resistance in the UA of fetuses with SUA decreases from the fetal end to the placental end. Knowing the normal values for fetuses with SUA might provide a better and more reliable Doppler ultrasound assessment.

## Data Availability

The datasets used and/or analysed during the current study available from the corresponding author on reasonable request.
